# Supplementary material for: RST1 and RIPR connect the cytosolic RNA exosome to the Ski complex in Arabidopsis
Source: Nat Commun. 2019 Aug 27;10:3871. doi: 10.1038/s41467-019-11807-4 (PMC6711988; doi:10.1038/s41467-019-11807-4)
Supplement: Supplementary file 1 — Supplementary Information [file 41467_2019_11807_MOESM1_ESM.pdf]

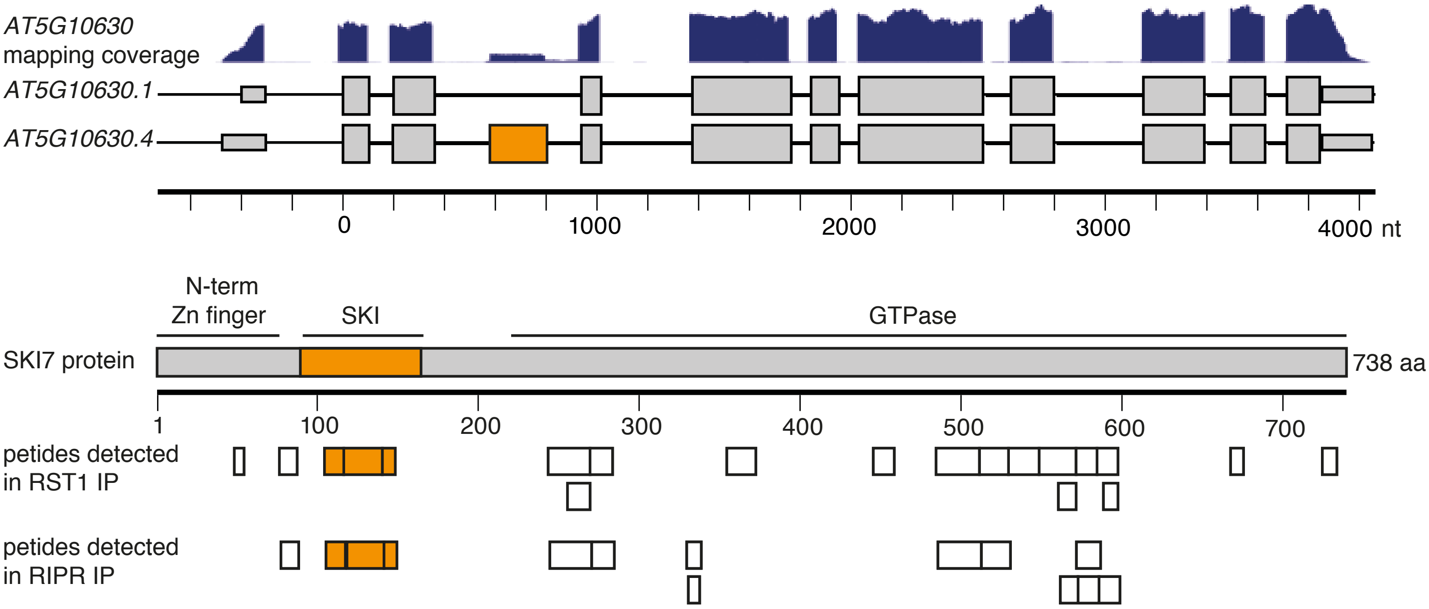

Supplementary Fig. 1 SKI7-specific peptides detected in RST1 and RIPR IPs. The top panel illustrates the mapping coverage (RNA seq data from light grown seedlings, source Araport11) of the *AT5G10630* locus and two (of five) gene models *AT5G10630.1* and *AT5G10630.4* encoding HBS1 and SKI7 proteins, respectively. Lines represent introns, small boxes represent 3' and 5' UTRs, large boxes represent exons. The exon specific to the SKI7- encoding mRNA is shown in orange. The lower panel illustrates the *Arabidopsis* SKI7 protein composed of an N-terminal and a Zn-finger domain thought to mediate the interaction with the SKI complex, the SKI7-specific domain encoded by the SKI7-specific exon and thought to be involved in recruitment of the exosome (in orange), and the large GTPase domain. The boxes below the diagram indicate unique peptides identified in the RST1 and RIPR co-immunoprecipitation experiments coupled to mass spectrometry. The SKI7-specific peptides are highlighted in orange.

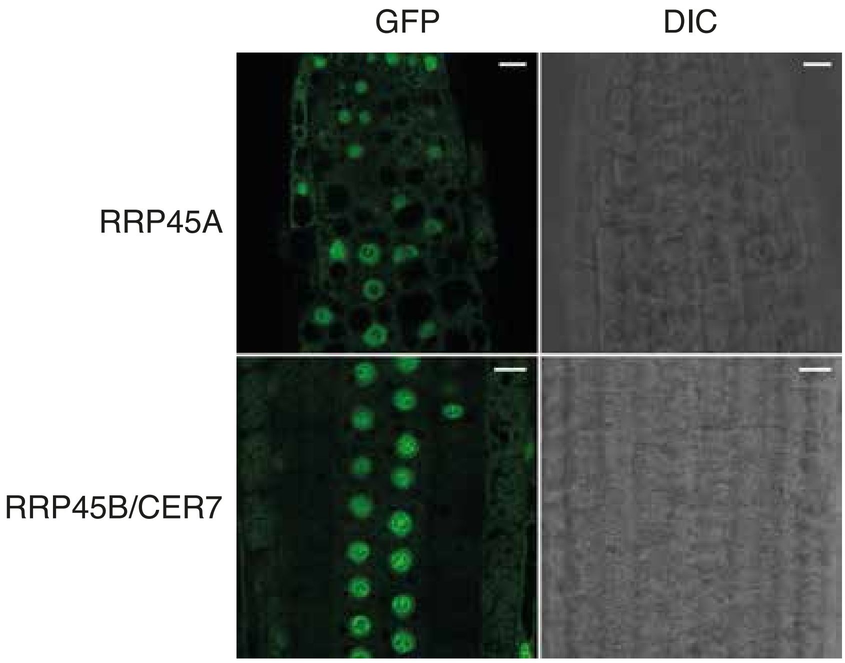

Supplementary Fig. 2 RRP45A and RRP45B/CER7 exosome subunits show similar intracellular distributions. Confocal microscopy images of *Arabidopsis* roots expression RRP45A (top) or RRP45B/CER7 (bottom) GFP fusion proteins under the control of the ubiquitin 10 promoter. DIC, differential interference contrast. Scalebars are 10µm.

predicted RIPR protein sequences

```
RIPR WT       1  MDSKSLAKSKRAHTLHHSKKSHSVHKPKVPGVSEKNPEKL  40
ripr{insT}    1  MDSKSLAKSKRAHTLHHSKKSHSVHKPKVPGVSEKNPEKL  40
ripr{insC}    1  MDSKSLAKSKRAHTLHHSKKSHSVHKPKVPGVSEKNPEKL  40

RIPR WT      41  QGNQTKSPVQSRRVSALPSNWDRYDDELDAAEDSSISLHS  80
ripr(insT)   41  QGNQTKSPVQSRRVSALPS*-------------------  60
ripr(insC)   41  QGNQTKSPVQSRRVSALPSQLGSV*--------------  65

RIPR WT      81  DVIVPKSKGADYLHLISEAQAESNSKIENNLDCLSSLDDL  120

RIPR WT     121  LHDEFSRVVGSMISARGEGILSWMEDDNFVVEEDGSGSYQ  160

RIPR WT     161  EPGFLSLNLNVLAKTLENVDLHERLYIDPDLLPLPELNTS  200

RIPR WT     201  QTKVSRNEEPSHSHIAQNDPIVVPGESSVREAESLDQVKD  240

RIPR WT     241  ILILTDESEKSSAIEADLDLLLNSFSEAHTQPNPVASASG  280

RIPR WT     281  KSSAFETELDSLLKSHSSTEQFNKPGNPSDQKIHMTGFND  320

RIPR WT     321  VLDDLLESTPVSIIPQSNQTSSKVLDDFDSWLDTI*  356
```

Supplementary Fig. 3 Frameshift mutations in *ripr(insT)* and *ripr(insC)* prevent the synthesis of full-length RIPR proteins.

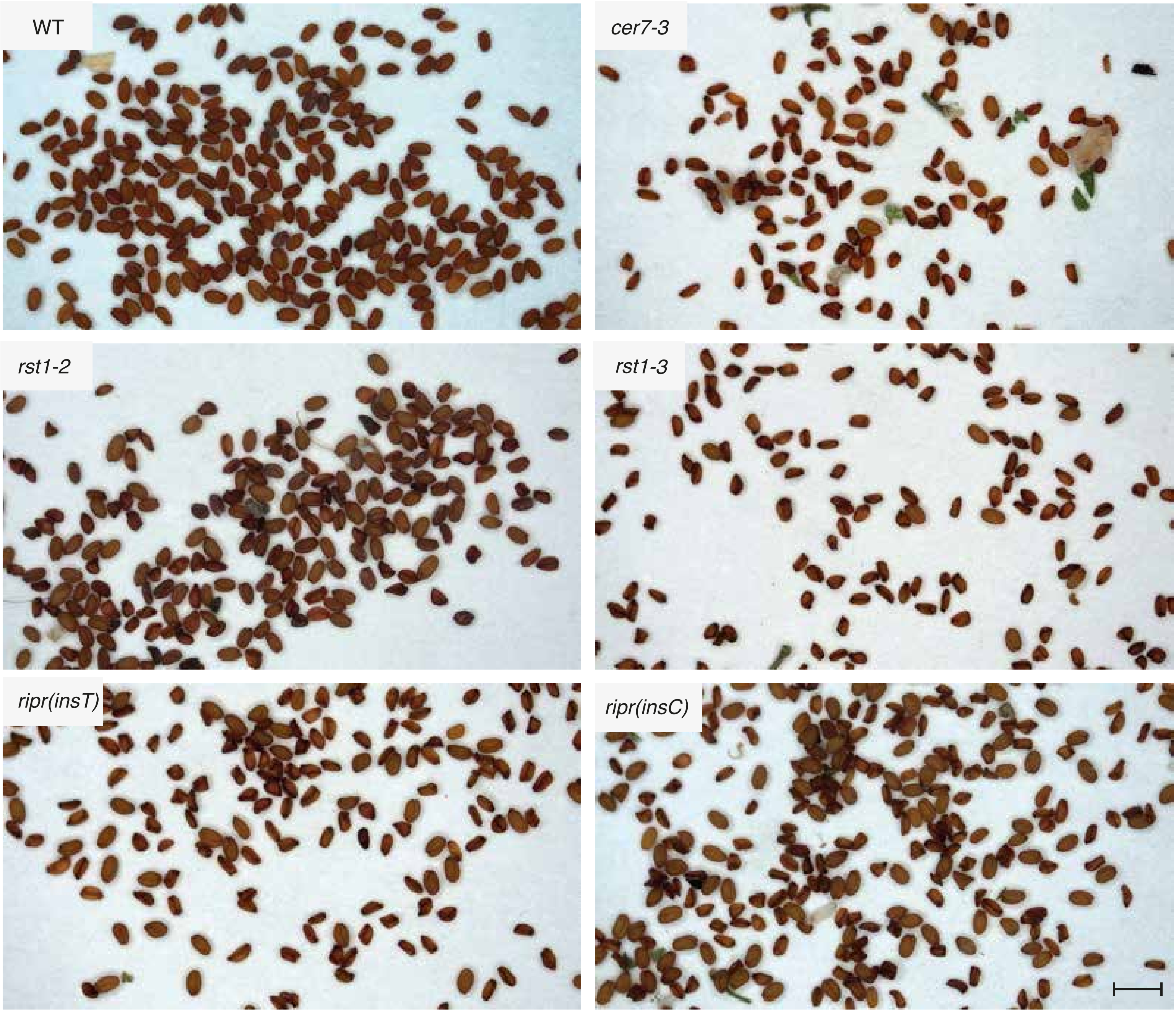

Supplementary Fig. 4 Seeds of Col-0 (WT), *cer7-3*, *rst1-2, rst1-3, ripr(insT)* and *ripr(insC)* plants. The mutants produce a high proportion of shrunken, inviable seeds. Scale bar is 1cm.

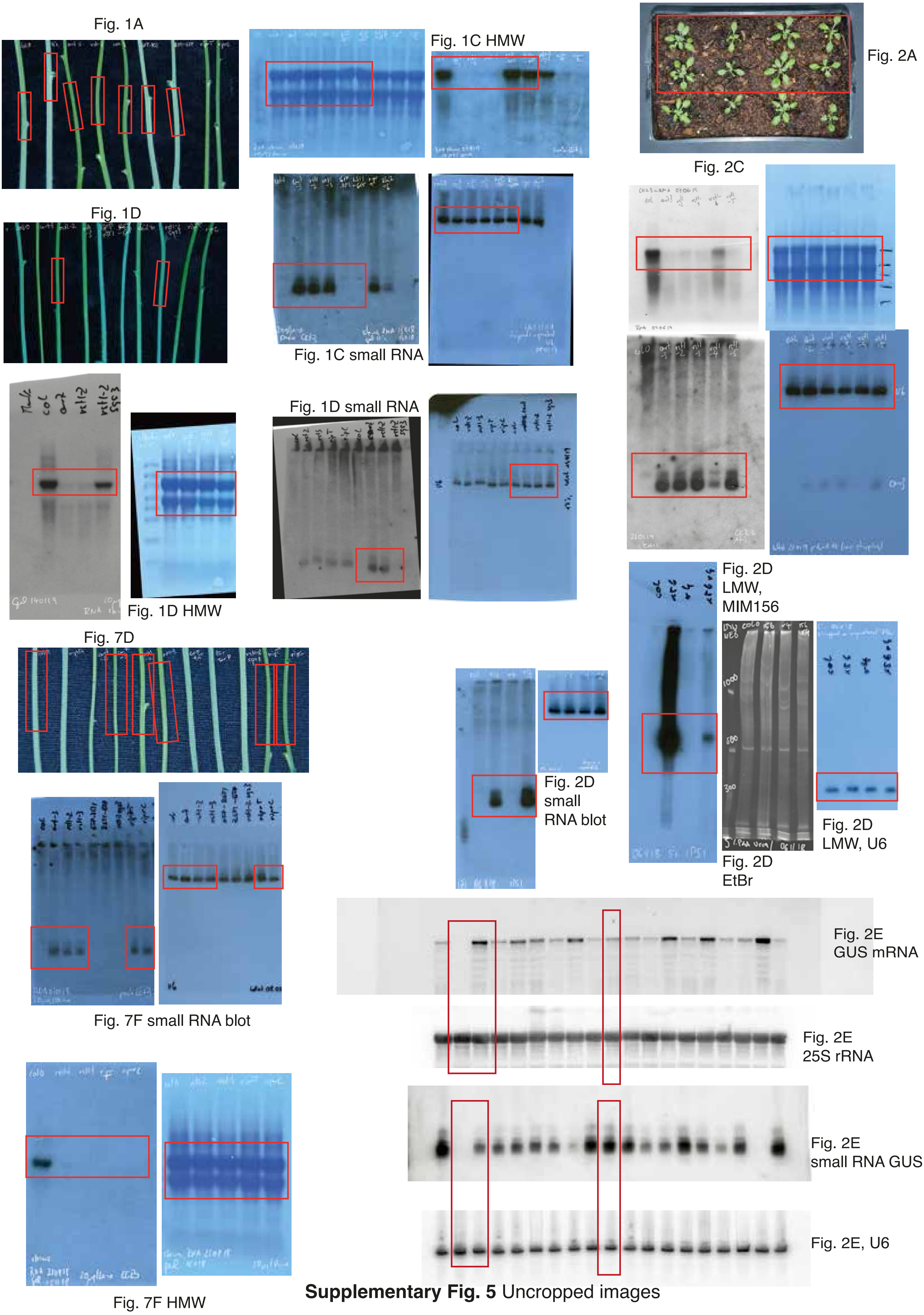

Supplementary Fig. 5 Uncropped images

Supplementary Table 1: Primers used in this study

Genotyping primers	
cer7-2 fw	ATATTTGAGTGGTGCTGCTGG
cer7-2 rev	AAACTCGACAAAGAGGGAAGC
cer7-3 fw	AAAGCTTCCCTCTTTGTCGAG
cer7-3 rev	GCCATTGGCATTAACTGTCAC
rrp45a fw	GTTGTTGGTTGCAGAGAAAGC
rrp45a rev	TGCGAGAAGTCTCAACATGTC
rst1-2 fw	GCGTGTTCTAAGCCATCTTTG
rst1-2 rev	GCAAGGAAATAAGAGCAAGGG
rst1-3 fw	TTGATTTCATCAATGGCTTCC
rst1-3 rev	CTGACAAGGGACGTTAGTTCG
rst1-4 fw	TGAGGTGTCTGAAGTGGTGCA
rst1-4 rev	CAAAGATGGCTTAGAACACGC, cleave product with *Ban1*
riprT/C fw	CGATGGACTCAAAATCTCTAGCTAAATCGAAGA
ripT/C rev	ACCTTGCCCGAACAACAAGA
sgs3-13 fw	AAGGCCATGCTTGTACATGAG
sgs3-13 rev	TATGAGGCTCTTAGAGCACGC
MIM156 fw	AAGAAAAATGGCCATCCCCTAGC
MIM156 rev	TGACAGAAGATAGAAGTGAGCAT
gRST1 fw	GACGTGTTGATTGAGATAGT
gRST1 rev	AACAGCTATGACCAT (M13 rev present in T-DNA)
probes	
CER3 fw	ACAGGTAATCTCAACTCCGAGG
CER3 rev	TGGAACACCAGCTACGACAC
IPS1 (MIM) fw	AAGAAAAATGGCCATCCCCTAGC
IPS1 (MIM) rev	TAGAGGGAGATAAACAAAACTCGCAGT
U6	GCTAATCTTCTCTGTATCGTTCCA
7SL	ATATGAAGATCGGACCAGCAGGC
