# Supplementary material for: RST1 and RIPR connect the cytosolic RNA exosome to the Ski complex in Arabidopsis
Source: Nat Commun. 2019 Aug 27;10:3871. doi: 10.1038/s41467-019-11807-4 (PMC6711988; doi:10.1038/s41467-019-11807-4)
Supplement: Supplementary file 3 — Description of Additional Supplementary Files [file 41467_2019_11807_MOESM3_ESM.pdf]

Description of Additional Supplementary Files

File Name: Supplementary Data 1
Description: Co-immunopurifications using RST1 as bait

File Name: Supplementary Data 2
Description: Co-immunopurifications using RRP41 as bait

File Name: Supplementary Data 3
Description: Co-immunopurifications using CER7 and RRP45A as bait

File Name: Supplementary Data 4
Description: Co-immunopurificatiosn using RIPR as bait

File Name: Supplementary Data 5
Description: Table of siRNAs enriched in cer7 samples as compared to WT
