# Supplementary material for: RST1 and RIPR connect the cytosolic RNA exosome to the Ski complex in Arabidopsis
Source: Nat Commun. 2019 Aug 27;10:3871. doi: 10.1038/s41467-019-11807-4 (PMC6711988; doi:10.1038/s41467-019-11807-4)
Supplement: Supplementary file 9 — Reporting Summary [file 41467_2019_11807_MOESM9_ESM.pdf]

nature research

Corresponding author(s): Dominique Gagliardi & Heike Lange

Last updated by author(s): Jul 29, 2019

Reporting Summary

Nature Research wishes to improve the reproducibility of the work that we publish. This form provides structure for consistency and transparency in reporting. For further information on Nature Research policies, see Authors & Referees and the Editorial Policy Checklist .

Statistics

For all statistical analyses, confirm that the following items are present in the figure legend, table legend, main text, or Methods section.

n/a	Confirmed	
☐	☒	The exact sample size (*n*) for each experimental group/condition, given as a discrete number and unit of measurement
☐	☒	A statement on whether measurements were taken from distinct samples or whether the same sample was measured repeatedly
☐	☒	The statistical test(s) used AND whether they are one- or two-sided<br>*Only common tests should be described solely by name; describe more complex techniques in the Methods section.*
☒	☐	A description of all covariates tested
☐	☒	A description of any assumptions or corrections, such as tests of normality and adjustment for multiple comparisons
☐	☒	A full description of the statistical parameters including central tendency (e.g. means) or other basic estimates (e.g. regression coefficient) AND variation (e.g. standard deviation) or associated estimates of uncertainty (e.g. confidence intervals)
☒	☐	For null hypothesis testing, the test statistic (e.g. *F*, *t*, *r*) with confidence intervals, effect sizes, degrees of freedom and *P* value noted<br>*Give P values as exact values whenever suitable.*
☒	☐	For Bayesian analysis, information on the choice of priors and Markov chain Monte Carlo settings
☒	☐	For hierarchical and complex designs, identification of the appropriate level for tests and full reporting of outcomes
☒	☐	Estimates of effect sizes (e.g. Cohen's *d*, Pearson's *r*), indicating how they were calculated

Our web collection on statistics for biologists contains articles on many of the points above.

Software and code

Policy information about availability of computer code

Data collection	GCMS-MS data acquisition: SCION TQ MS Workstation (Bruker) v8.2.1<br>LCMS-MS data acquisition: Xcalibur (Thermo) v4.1.31.9; Raw data processing: Proteome Discoverer (Thermo) v2.0.0.802; Peptide and protein identification (database search): Mascot (Matrix Science) v2.5, Peptide and protein validation, Spectral Count quantification and samples alignment: Proline (ProFI consortium) v 1.4.<br>RNA sequencing: HiSeq 4000, HCS HD (Illumina) v3.4.0
Data analysis	GCMS-MS: peak identification and comparison of mass spectra to databases, MS Data Review (Bruker) v8.2<br>Statistical analysis of LCMS-MS data (R-studio v1.1.453): scale factor calculation, DESeq2 v1.12.4; generalized linear model for p-value calculation, edgeRv3.14.0; p-value correction, p.adjust function from Stats v3.3.1; MDS plots: dist and cmdscale functions from R Stats v3.3.1; plot function from graphics v3.3.1; annotation: BiomartR v2.28.0; volcano plots: ggplot2 v3.1.0<br>RNA sequence analysis (R v3.5): trimming of reads, cutadapt v1.18; mapping to Tair10, ShortStack v3.8.5; differential expression analysis, DESeq2 v1.22.1; venn diagram: serial list v 2.3 and https://www.meta-chart.com/venn#/display.

For manuscripts utilizing custom algorithms or software that are central to the research but not yet described in published literature, software must be made available to editors/reviewers. We strongly encourage code deposition in a community repository (e.g. GitHub). See the Nature Research guidelines for submitting code & software for further information.

Data

Policy information about availability of data

All manuscripts must include a data availability statement. This statement should provide the following information, where applicable:

- Accession codes, unique identifiers, or web links for publicly available datasets
- A list of figures that have associated raw data
- A description of any restrictions on data availability

Gel and blot images
Uncropped blots and stem images are provided in Supplementary Figure S5.

The small RNAseq and mass spectrometry proteomics raw data that support the findings of this study have been deposited to the NCBI Gene Expression Omnibus (GEO) database, accession code GSE129736 [https://www-ncbi-nlm-nih-gov.insb.bib.cnrs.fr/geo/query/acc.cgi?acc=GSE129736], and to the ProteomeXchange Consortium via the PRIDE75 partner repository with the dataset identifier PXD013435, [https://www.ebi.ac.uk/pride/archive/projects/PXD013435], respectively. Full resolution versions of all images, the wax analysis data, the processed small RNA-seq data and interactive volcano blots are available at figshare [https://doi.org/10.6084/m9.figshare.c.4483406]

Field-specific reporting

Please select the one below that is the best fit for your research. If you are not sure, read the appropriate sections before making your selection.

☒ Life sciences ☐ Behavioural & social sciences ☐ Ecological, evolutionary & environmental sciences

For a reference copy of the document with all sections, see nature.com/documents/nr-reporting-summary-flat.pdf

Life sciences study design

All studies must disclose on these points even when the disclosure is negative.

Sample size	RNA and protein samples were prepared from pooled stem sections or flowers collected from 5-10 plants grown at the same time in identical conditions. For the GCMS-MS, three stem sections from three plants were analysed. This sample size is sufficient to ensure that variation among individual plants has no significant influence on the result.
Data exclusions	No data were excluded from the analysis.
Replication	Biological replicates are defined as plants of the same genotype grown at different times. In addition, some biological replicates originated from a different seed stock or an independent mutagenesis event. For RNA blots, at least three biological replicates were analysed. A representative experiment is shown in each figure. For analysis of the wax-deficient phenotype, pictures were taken from three biological replicates. Three plants of one replicate were further analysed by GCMS-MS. For microscopy, at least two independent transformants were examined for each construct. For RNA sequencing, two independent alleles for each mutation and three biological replicates for each genotype (two for cer7-4) were analysed together. The precise number of biological and technical replicates used for each of the co-immunopurification experiments is provided in the material and method section.
Randomization	Typically we grow 20-40 plant per genotype and replicate, 5-10 of which are used for flower harvesting. Selection is random except that plants obviously affected by greenhouse pests are omitted.
Blinding	none

Reporting for specific materials, systems and methods

We require information from authors about some types of materials, experimental systems and methods used in many studies. Here, indicate whether each material, system or method listed is relevant to your study. If you are not sure if a list item applies to your research, read the appropriate section before selecting a response.

Materials & experimental systems

n/a	Involved in the study	
☒	☐	Antibodies
☒	☐	Eukaryotic cell lines
☒	☐	Palaeontology
☒	☐	Animals and other organisms
☒	☐	Human research participants
☒	☐	Clinical data

Methods

n/a	Involved in the study	
☒	☐	ChIP-seq
☒	☐	Flow cytometry
☒	☐	MRI-based neuroimaging
